# Intraoperative parameters and postoperative follow-up of foam-based intraperitoneal chemotherapy (FBIC)

**DOI:** 10.3389/fphar.2023.1276759

**Published:** 2023-11-14

**Authors:** Carolina Khosrawipour, Jakub Nicpoń, Zdzisław Kiełbowicz, Przemysław Prządka, Bartłomiej Liszka, Said Al-Jundi, Veria Khosrawipour, Shiri Li, Hien Lau, Joanna Kulas, Agata Diakun, Wojciech Kielan, Mariusz Chabowski, Agata Mikolajczyk-Martinez

**Affiliations:** ^1^ Faculty of Medicine, Wroclaw Medical University, Wroclaw, Poland; ^2^ Department of Surgery, Faculty of Veterinary Medicine, Wroclaw University of Environmental and Life Sciences, Wroclaw, Poland; ^3^ Department of Surgery, Petrus-Hospital Wuppertal, Teaching Hospital of the Medical University Düsseldorf, Wuppertal, Germany; ^4^ Division of Colon and Rectal Surgery, Department of Surgery, New York Presbyterian Hospital, Weill-Cornell College of Medicine, New York, NY, United States; ^5^ Department of Surgery, University of California-Irvine (UCI), Irvine, CA, United States; ^6^ Faculty of Veterinary Medicine, Wroclaw University of Environmental and Life Sciences, Wroclaw, Poland; ^7^ 2nd Department of General Surgery and Surgical Oncology, Wroclaw Medical University, Wroclaw, Poland; ^8^ Department of Surgery, 4th Military Hospital, Wroclaw, Poland; ^9^ Faculty of Medicine, Wroclaw University of Science and Technology, Wroclaw, Poland; ^10^ Department of Biochemistry and Molecular Biology, Faculty of Veterinary Sciences, Wroclaw University of Environmental and Life Sciences, Wroclaw, Poland

**Keywords:** foam-based intraperitoneal chemotherapy, chemotherapy, doxorubicin, peritoneal metastasis, laparoscopy

## Abstract

**Background:** For decades, intraperitoneal chemotherapy (IPC) has been delivered into the abdominal cavity as a liquid solution. Recently the concept of foam as a carrier-solution for IPC was suggested. This *in-vivo* swine study aims to evaluate the safety, intraoperative parameters, limitations and postoperative complications of foam-based intraperitoneal chemotherapy (FBIC).

**Methods:** Three 65-day-old swine received FBIC with doxorubicin in a laparoscopy setting. Intraoperative parameters were monitored throughout the procedure and an extensive postoperative laboratory monitoring was conducted for 7 days. At day seven an autopsy was performed for further evaluation.

**Results:** The insufflation of FBIC caused a temporary rise in blood pressure and a simultaneous drop in heart rate. Capnography detected a continuous increase in end-tital CO_2_ levels. A temporary drop of intraabdominal temperature was noted. Postoperative blood and serum laboratory results did not indicate any organ failure. No indication of intraperitoneal infections was noted and no structural tissue changes were visible in the autopsy.

**Discussion:** The application of FBIC appears to be a feasible approach regarding intraoperative anesthesiology and postoperative surgical management. A lack of postoperative structural changes on the seventh day were a promising sign of safety and biocompatibility. Surgical reintervention would have been possible. To discuss a possible clinical application, further studies are required to investigate long-term safety, pharmacodynamics and the antitumoral potential of FBIC.

## 1 Introduction

Decades of clinical and experimental research on the management of Peritoneal Metastasis (PM) have not significantly changed the overall poor prognosis rate. Due to the rapid progression of PM median survival rates are only about 3–4 months (Sadeghi et al., 2000; Goéré et al., 2017; Gelli et al., 2018). Patients suffering from PM endure a lot of morbidities. Significant contributions have been made in an attempt to improve the outcome of peritoneal surface malignancies. The current research focuses on a wide variety of topics such as molecular biology (Cheng et al., 2019; Shao et al., 2023), pharmacological enhancements (Schubert et al., 2019; Khosrawipour T. et al., 2020; Mikolajczyk et al., 2022) and clinically established concepts like HIPEC and cytoreductive surgery (Souadka et al., 2022; Patel et al., 2023). One of the main areas of PM research is the improvement and the better understanding of locoregional and intraperitoneal chemotherapy (IPC).

In the previous decades IPC has been delivered into the abdominal cavity as a liquid solution. However, a range of limitations were witnessed when “classic” liquid chemo solutions were used. Therefore, Pressurized intraperitoneal aerosol chemotherapy (PIPAC) had been proposed as a new and improved concept (Khosrawipour et al., 2018; Khosrawipour V. et al., 2020). Since then, the delivery of aerosolized chemotherapy has been used for a selected groups of patients who would otherwise not qualify for Hyperthermic intraperitoneal chemotherapy (HIPEC) and Cytoreductive surgery (CRS) (Khosrawipour et al., 2017). The advantage of PIPAC delivery is due to the physics behind its aerosolized application method (Göhler et al., 2017; Mikolajczyk et al., 2018a; Khosrawipour et al., 2019). Furthermore, a large amount of research has been conducted to try to further improve this technology (Lau et al., 2020; Diakun et al., 2022a; Diakun et al., 2022b; Diakun et al., 2022c; Hwang et al., 2022).

Foam has some unique characteristics. Some possible advantages of foam are slow degradation which allows for an extended drug contact time in the peritoneum. The expansion of foam is multidirectional and more homogenous. This means that the contact of a concentrated drug with peritoneal tissue can be expanded without diluting drug volume. Foam-based intraperitoneal chemotherapy (FBIC) could be a feasible option for the treatment of PM. Therefore, it has been proposed as a drug carrier for intraperitoneal chemo applications (Schubert et al., 2020). Our *in-vivo* study aims to evaluate FBIC as a new vehicle for IPC delivery. The focus of this study is about the consequences on the cardiovascular system, pulmonary system and the postoperative laboratory parameters. The effects of FBIC will be evaluated by postoperative data which will also be helpful for further clinical application.

## 2 Methods

### 2.1 The laparoscopic *in-vivo* swine model

**FIGURE 1:**
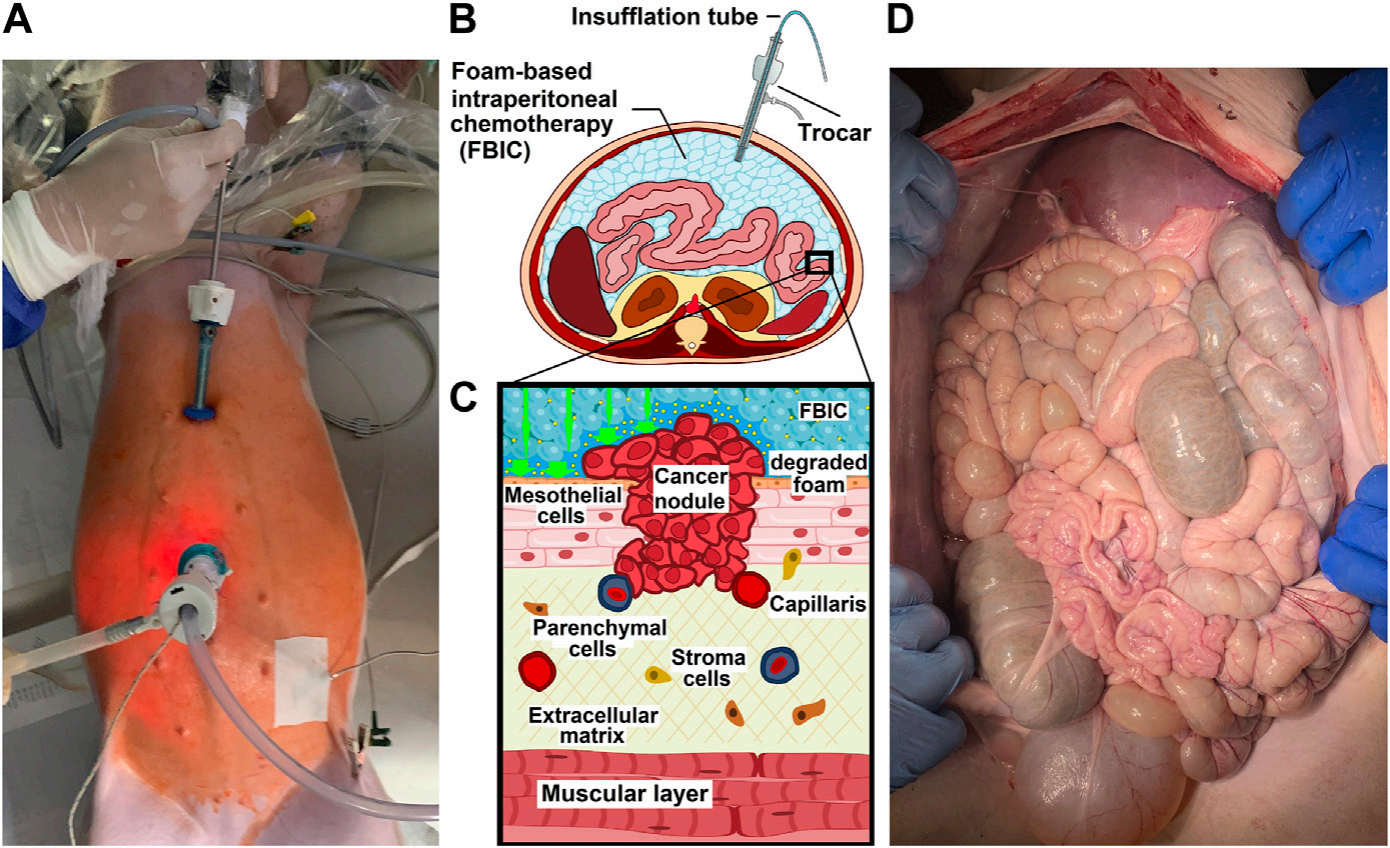
**(A)**. Birds eye view onto a *in-vivo* laparoscopy while foam based intraperitoneal chemotherapy (FBIC) is applied. Disinfected operative field is colored red (iodine). Surgical entrance site is visible: a large 10mm trocar is placed periumbilical and a small 5 mm trocar is placed epigastral. Additional temperature sensors are placed to monitor temperature development during the procedure. **(B)**. Transversal section of FBIC delivery. **(C)**. Model of surface interaction of FBIC in the peritoneum. FBIC degradation on the peritoneal surface with highly concentrated doxorubicin fluid-film (large green-arrow). **(D)**. Birds eye view: laparotomy and exploration after autopsy at postoperative day 7.

The study includes three 65-day-old swine. A laparoscopy was planned, all swine were prepared for anesthesia and ventilation. The swine received an intramuscular injection of midazolam (0.3 mg/kg, WZF Polfa S.A., Warsaw, Poland), medetomidine (0.02 mg/kg, Cepetor 1 mg/mL, CP-Pharma Handelsgesellschaft, Burgdorf, Germany) and ketamine (9 mg/kg, Ketamine 100 mg/mL, Biowet Puławy sp. z o.o., Warsaw, Poland). Additionally, all swine received analgesia with propofol at 1 mg/kg. The swine were intubated, and anesthesia was continued with isoflurane 1%. Additional analgesia was provided with fentanyl 2 µg/kg. A continuous intravenous fluid line was established, and crystalloid fluid was given at 0,2–0,3 µg/kg/min. Before surgery all swine were placed in a supine position. An infra-umbilical mini laparotomy was performed. A 10 mm trocar (Kii®Balloon Blunt Tip System, Applied Medical, Rancho Santa Margarita, CA, USA) was inserted through the mini laparotomy. Under visual guidance A 5 mm trocar was placed at a distance to the first trocar. The abdominal cavity was insufflated with CO_2_ to maintain a capnoperitoneum (Olympus UHI-3 insufflator, Olympus medical life science and industrial divisions, Olympus, Shinjuku, Tokio Japan). A full diagnostic laparoscopy was performed using a 5 mm camera system (Karl Storz 5mm/30° Laparoscope/Tuttlingen, Germany) (Figures 1A–C). After visual confirmation of no pathologies the “foam-insufflation” tube was introduced into the 10 mm trocar. After the tube was inserted and its positioning was confirmed via visual imaging. Afterwards the laparoscope was removed. Then a temperature probe was inserted through the trocar into the abdomen. The CO_2_ from the capnoperitoneum was evacuated. For the measurement of the central body temperature, a temperature probe was placed in the esophagus. Another probe was placed outside the abdomen fixed with adhesive tape. An invasive arterial pressure line was placed and ECG electrodes monitored the heart rate.

### 2.2 Postoperative monitoring

One operative procedure was performed per day. All swine were kept together and were monitored for the next 7 days for behavior changes, feeding habits, indication of pain and surgical site infection. At postoperative days 1, 3 and 7 (1 d, 3 d, 7 d) blood was taken for a blood count and serological measurements. On the last postoperative day (7 d) an autopsy was performed (Figure 1D).

### 2.3 Euthanization

The swine were premedicated with an intramuscular injection of midazolam (0.1 mg/kg, Midanium 5 mg/mL), medetomidine (0.02 mg/kg, Cepetor 1 mg/mL) and ketamine (8 mg/kg, Ketamine 100 mg/mL) mixture. After that, they were euthanized according to recommendations (Garber et al., 2011) with an intravenous injection by Sodium Pentobarbital with Pentobarbital (50 mg/kg with 12 mg/kg, Morbital 133,3 mg/mL + 26,7 mg/mL).

### 2.4 The bicarbonate-based foam carrier

The ratio of foam ingredients for the bicarbonate-foam were mathematically and experimentally predetermined. The basic chemical reactions were analyzed and quantified in order to determine the most suitable molar combination. The major components of foam are citric acid (Sigma-Aldrich, St. Louis, USA) and sodium bicarbonate (Sigma-Aldrich, St. Louis, USA). Doxorubicin hydrochloride (PFS^®^, 2 mg/mL, Pfizer, Sandwich, United Kingdom) was added as a chemotherapeutic component (1,5 mg/meter^2^ body surface). Furthermore, iodide-based contrast media was used for CT (AccupaqueTM 350 mg J/ml, GE Healthcare, Chicago USA).

### 2.5 Statistical analyses

Experiments were independently performed. The statistical analyses were done with GraphPad Prism [GraphPad Software Inc., version 8.0.2 (263)]. Descriptive statistics included mean, median and percentiles. Probability (p) values were calculated via one factorial ANOVA (parametric) test results include: *p < 0.05 and **p < 0.005, and #*p* > 0.05, with *p*-value <0.05 considered to be statistically significant.

### 2.6 Ethical approval and regulations

An Approval of the Local Board on Animal Care was obtained for the *in-vivo* swine experiments (UCHWALA NR 029/2021/P1) according to Polish regulations and European Union law. The data in the study displays a multi-stage study on *in-vivo* FBIC.

## 3 Results

The *in-vivo* experiments on the three swine were successfully conducted. No intraoperative or postoperative complications were detected. No major anesthesiologic complications were observed. There was no indication of anaphylaxis or cardiovascular collapse and no problems concerning intubation or extubation of the swine occurred. No (general) hypothermia or respiratory depression was noted. 30 min into the procedure all swine were successfully extubated. 20 min after insufflation a camera was inserted into the peritoneum in order to visualize the abdominal cavity. The intraabdominal foam did not interfere with the diagnostic overview. After a total of 30 min the trocars were removed and the mini laparotomy was sutured. No surgical problems or complications were observed during and after surgery. All animals survived the following postsurgical recovery. After the procedure all swine drank and ate adequately. No pain or behavioral changes were observed during the days of recovery.

### 3.1 Development of central body temperature, abdominal cavity and skin temperature

**FIGURE 2:**
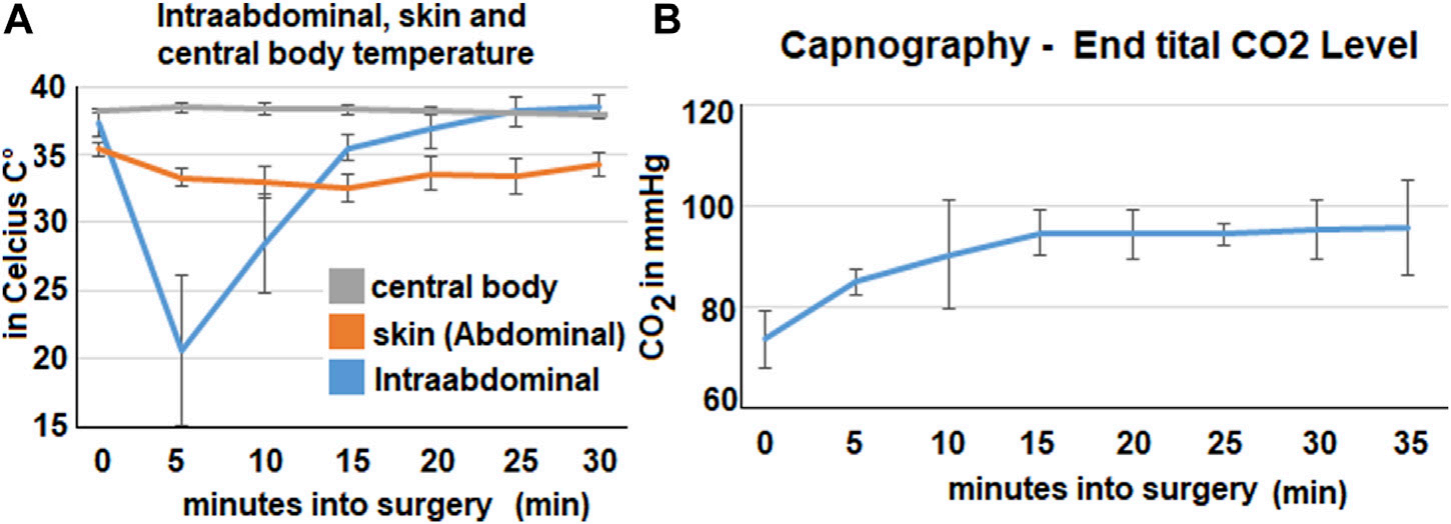
Intraoperative data from temperature probes, capnometry and respiratory rate. The mean values calculated from all three swine are represented by the data points. **(A)**. Data from the three temperature probes at various locations: skin probe on the abdomen, intraabdominal probe and central body temperature (esophageal probe). **(B)**. Expiratory CO_2_ -levels in the capnometry in mmHg.

Within the cavity a rapid reduction of temperature can be observed during the insufflation of the bicarbonate foam. Within the first 5 min the medium temperature drops down to 20,6°Celsius and then steadily increases again (Figure 2A). After 15 min, it is above 35°Celsius. During the procedure the skin temperature on the abdomen decreases. It reaches its lowest point 15 min into the procedure when it drops to 32,5°Celcius (mean). After that it increases slowly and reaches around 35,4°Celsius (mean). During the whole procedure the central body temperature remains stable and does not change significantly.

The capnography shows an increase in mean end-tital CO_2_ (Figure 2B). The initial level of 74 mmHg CO_2_ increases to around 94–95 mmHg. This increase happens within the first 15 min and plateaus afterwards.

### 3.2 Results on heart rate and blood pressure

**FIGURE 3:**
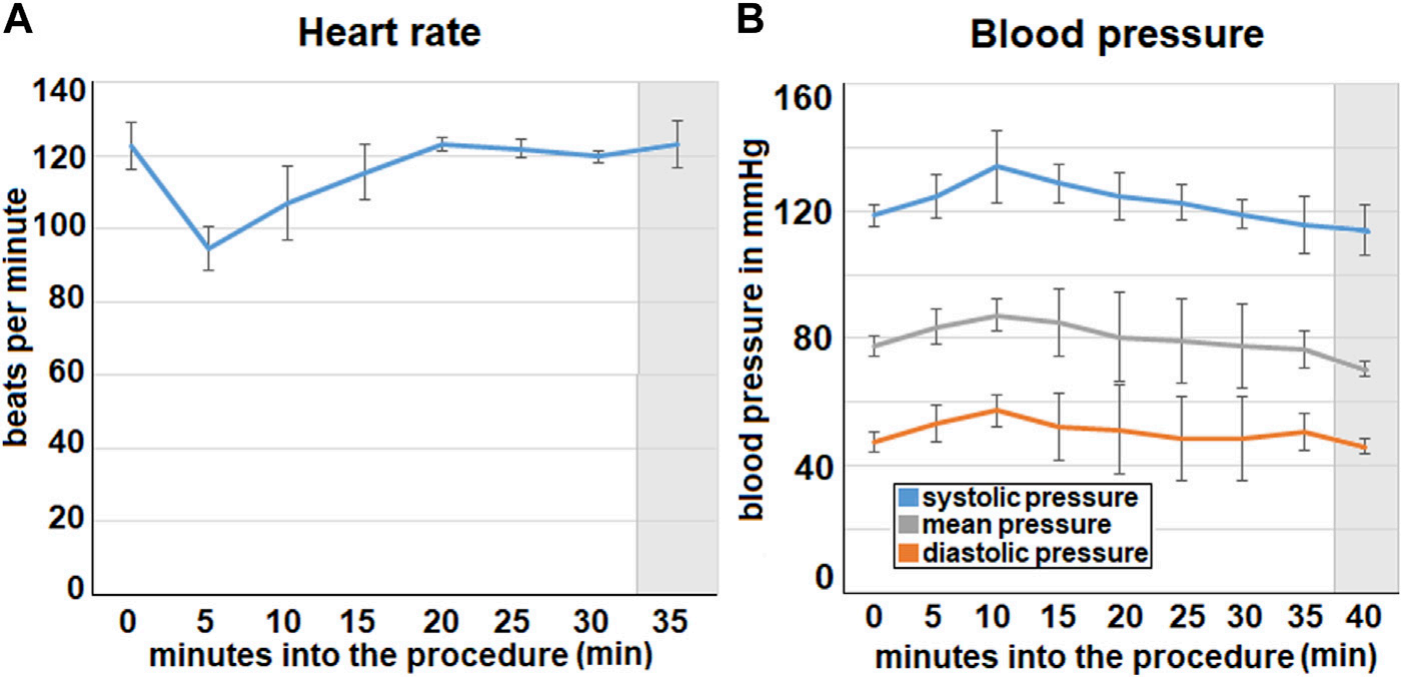
Intraoperative data concerning **(A)**. heart rate and **(B)**. blood pressure (via invasive measurement). The mean values calculated from all three swine are represented by the data points. **(A)**. Mean heart rate from three swine with the standard deviation. **(B)**. Mean blood pressure from all three swine with standard deviation. Extubating phase and postoperative monitoring are marked in grey.

In the first 5 min of the procedure the mean heart rate decreases from a baseline of 123 ± 6 beats per minutes down to 95 ± 6. 20 min into the procedure the heart rate then increases again and reaches its initial level (Figure 3). After this the heart rate remains stable.

The blood pressure increases from the beginning of the foam insufflation and reaches its peak at about 10 min into the procedure where it is 134 ± 12 mmHg. After this the pressure declines continuously. This can be observed for the systolic, diastolic and the mean blood pressure.

### 3.3 Development of intraoperative and postoperative blood count

**FIGURE 4:**
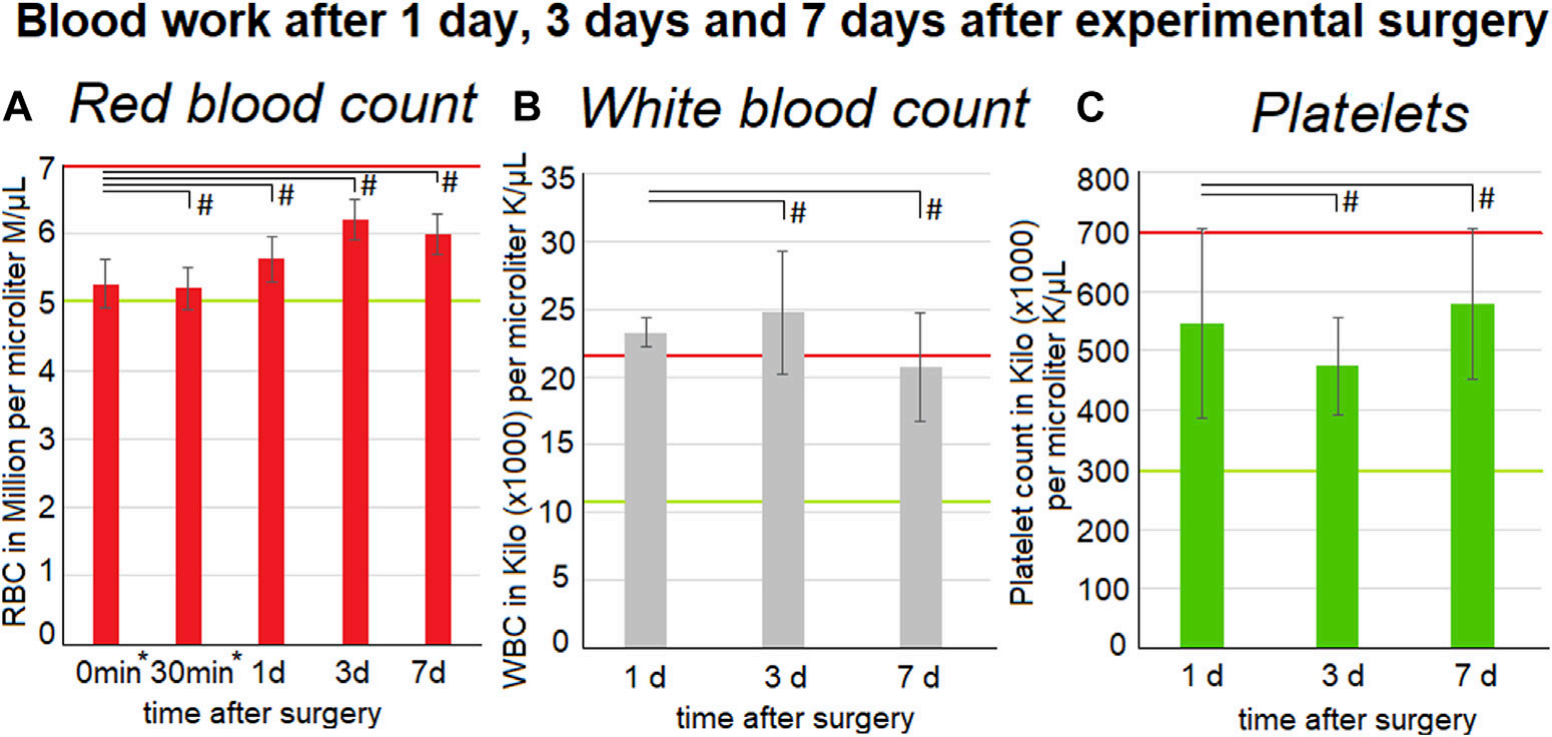
Blood work-up one, three and 7 days after surgery. The mean values calculated from all three swine are represented by the data points. Listed below are red blood cell count (left) **(A)** inclusive intraoperative measurements at 0* and 30* minutes into surgery and also white blood cell count (middle) **(B)** and platelet count (right) **(C)**. Medium and standard deviations are presented. Red and green lines indicate the normal reference levels (95% Interval - red: upper limited and green: lower limit) for the parameters. The *p* values are indicated as *p < 0.05, **p < 0.005, and #*p* > 0.05, with *p*-value <0.05 considered to be statistically significant.

The red blood count could be gathered form the intraoperative measurements at 0 min and at 30 min into the procedure. Furthermore, data was gathered on the first, third and seventh postoperative day. The red blood count did not change and remained within the reference levels of above 5 million per microliter. The white blood count remains at the upper reference level of around 22.000 per microliter. Although no significant changes could be observed there was a peak within the values on the third(3 d) postoperative day (Figures 4A–C). The postoperative platelet count varied a lot on the first (1 d) (5,5 ± 1,6) ×106/µl, 3 d (4,7 ±0,8) ×106/µl and seventh day (7d) (5,8 ± 1,3) ×106/µl. The platelet count did not exceed the upper reference which is around 3-7 ×106/µl.

### 3.4 Development of postoperative serum parameters

**FIGURE 5:**
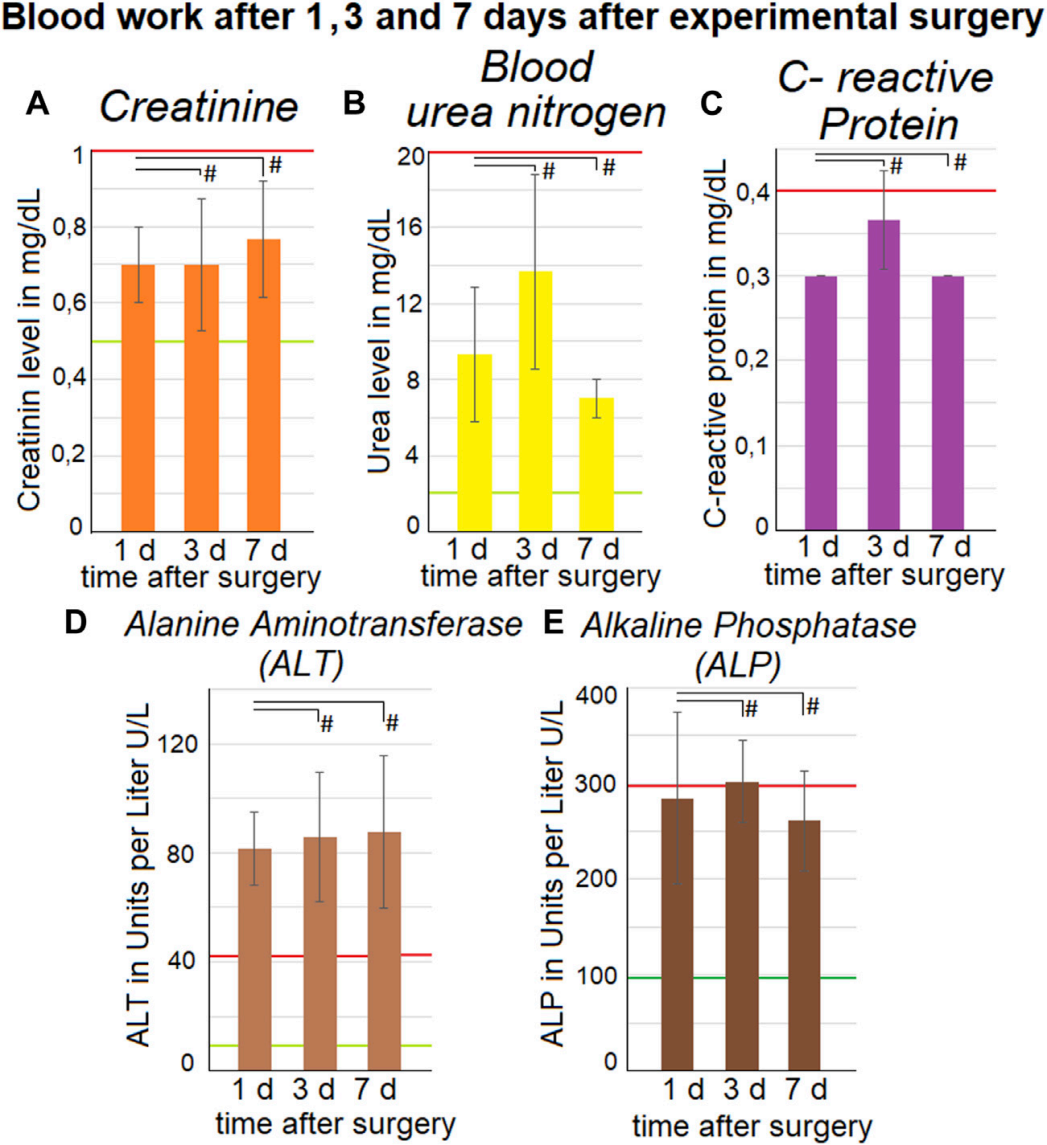
Blood work-up one, three and 7 days after surgery. The mean values calculated from all three swine are represented by the data points. Upper-section: listed are **(A)**. creatinine, **(B)**. blood urea level and **(C)**. C-reactive protein. Medium and standard deviations are presented. Liver parameters are listed in the lower section with **(D)**. alanine aminotransferase (ALT) and **(E)**. Alkaline Phosphatase (ALP). Mean and standard deviations are presented. Green lines indicate the lower limit and red lines indicate the upper limit for the parameters. The *p* values are indicated as *p < 0.05, **p < 0.005, and #*p* > 0.05, with *p*-value <0.05 considered to be statistically significant.

The kidney related parameters remained mostly stable. The creatinine level did not change significantly (*p* < 0.05) from the first day 0,7 ± 0,1 mg/dL to 7d 0,77 ± 0,15 mg/dL although a slight mean increase was noted. The maximum level for creatinine at 2,1 mg/dL was not reached (Figures 5A–C). The blood urea levels peaked on the third day with 13,7 ± 5,1 mg/dL but decreased again on the seventh day with 7 ± 1 mg/dL. The maximum physiological level for blood urea 30 mg/dl was not reached. While the white blood count was slightly above the upper reference range, the levels of C-reactive protein remained below the upper reference limit of ≤ 0,4 mg/dL. Therefore, no indications of an extensive tissue infection were present.

### 3.5 Development of postoperative liver parameters

The blood levels of two liver enzymes were measured. One was Alanine Aminotransferase (ALT) and the other one was Alkaline Phosphatase (ALP) (Figures 5D, E). The mean ALT level was at 81,7 ± 13,3 U/L for the first day, 85,7 ± 23,6 U/L for the third day and 87,7 ± 28,1 U/L for the seventh day. During these 3 days the ALT level was stable but still higher than the refence range of 9–43 U/L. No levels of ALT were available from before the procedure, so no comparison was possible.

The ALP level was at 284,7 ± 89,2 U/L for the first day, 302 ± 43,2 U/L for the third day and 260,7 ± 51,7 U/L for the seventh day. During all 3 days the levels of ALP were close to the upper reference level of 294 U/L. There is no indication of an increase or decrease of ALP levels during the observed interval. For the ALP no preoperative levels were available, so a comparison was not possible.

## 4 Discussion

The intraperitoneal administration of chemotherapeutic solution is an established treatment of PM. The local instillation of chemotherapeutic solution increases cell toxicity when it is in direct contact with the cancer nodules of the peritoneal cavity. Even though extensive attempts have been made to improve IPC delivery (Mikolajczyk et al., 2020; Buggisch et al., 2022; Khosrawipour et al., 2023a) limitations still exist in both liquid instillations as well as aerosol-based systems (Dedrick and Flessner, 1997; Sugarbaker and Ryan, 2012; Khosrawipour et al., 2016a; Bellendorf et al., 2018). These limitations include inhomogeneous drug distribution and limited penetration into PM. Novel substances have also been used for intraperitoneal delivery however some show limited drug tissue penetration (Mikolajczyk et al., 2018b). Therefore, new delivery methods have been presented to improve IPC (Buggisch et al., 2022; Hwang et al., 2022). For the patients to have a better outcome, new physical methods of drug delivery have been used to enhance the antitumoral effect of IPC (Khosrawipour et al., 2016b; Khosrawipour et al., 2023a). One of these new physical methods is FBIC (Schubert et al., 2020).

In this study we were able to demonstrate that FBIC is a feasible concept. The data on intraoperative vital parameters, capnography, body temperature and respiratory rate indicate that the insufflation of FBIC is compatible with total anesthesia under external ventilation. Analyses of the postoperative blood work reveal no major changes on the red and white blood cell count and no changes on the platelet cell count. The postoperative time was limited to 7 days which shortened the observation of pathologies in the swine. However, within this period the large exposure to citric acid did not cause liver tissue necrosis.

Although the ALT was slightly above the upper reference range, ALP levels were not increased. This observation is relevant for FBIC because it shows that there is only limited damage to the liver. Citric acid at higher dosages can cause oxidative damage of hepatocytes by decreasing the activity of antioxidative enzymes. This has been studied on *in-vivo* mice models after intraperitoneal delivery with exposure to large amounts of citric acid (120–480 mg/kg-body weight) (Chen et al., 2014). However, no final conclusions regarding liver toxicity could be made because preoperative levels of ALT and ALP were not available. This needs to be reexamined in follow up studies.

CRP and white blood count show no signs of infection or inflammation. This is highly relevant as FBIC could have induced local inflammation, perforation or necrosis on large surfaces such as the peritoneum. This is important because of the effects that barium sulfate can have when it gets into contact with the peritoneal cavity. Although barium sulfate is a safe and non-toxic substance for oral intake it causes severe inflammation (Williams and Harned, 1991) upon entering the peritoneal cavity.

At the beginning, it was not possible to exclude a similar reaction on the peritoneal surface with the intraperitoneal delivery of FBIC. Due to this, it was important to evaluate the interaction of FBIC with the peritoneal surface. Therefore, on the seventh day the swine were autopsied. A thorough examination revealed no intraabdominal macroscopic pathologies nor signs of “changes” in the peritoneum (Figure 1D). This was also supported by histopathological analyses of the peritoneal tissue at various locations which is currently studied (Khosrawipour et al., 2023b). The core body temperature did not change even though the intraabdominal temperature decreased. At this point it is important to mention that the chemical reaction of bicarbonate and citric acid is endotherm. Looking forward we want to establish a better understanding of the temperature and distribution profile of FBIC. These aspects have been studied for the HIPEC in more detail (Cianc et al., 2018).

However, based on the presented data we can assume that the bicarbonate carrier system does not cause significant local toxicity and inflammation.

Intraoperative and early postoperative systemic effects of FBIC could potentially be more critical than concerns over local tissue biocompatibility.

The limited number of swine for the pilot project were set by the animal ethic commission. We hope that the current results will enable us to receive approval for a larger study series where more parameters can be analyzed. The postoperative observation was limited to 7 days. Increasing the overall observation time to 30 days or even 6 weeks would be helpful for a more substantial evaluation of the procedure. However, the combination of FBIC with a surgical procedure such as partial intestinal resection with anastomosis is feasible and could be considered in a follow-up study.

After further studies potential systemic effects could be addressed. However, it is very promising that we do not observe any adverse reactions 7 days into the procedure. Although we only had three swine in this study no postoperative complications and kidney, liver and pulmonary failures could be observed 7 days postoperatively.

The safety of this method needs to be confirmed by other studies involving more subjects. The evaluation of toxicity in an *in-vivo* setting and the pharmacological and antitumoral potentials of FBIC must be studied as well.

## 5 Conclusion

The unique characteristics of foam might be significantly advantageous for IPC treatment in PM. From an anesthesiologic perspective, if intraperitoneal foam is applied the challenges involving abdominal expansion and intraperitoneal degradation are manageable. The early postoperative organ functions do not seem to be critically impaired by the particular carrier-system. Although the current results are encouraging further research needs to be conducted to evaluate pharmacological and antitumoral potentials of FBIC as well as safety beyond the postoperative period of 7 days.

## Data Availability

The original contributions presented in the study are included in the article, further inquiries can be directed to the corresponding author.
